# The Actuation System of the Ankle Exoskeleton T-FLEX: First Use Experimental Validation in People with Stroke

**DOI:** 10.3390/brainsci11040412

**Published:** 2021-03-24

**Authors:** Daniel Gomez-Vargas, Felipe Ballen-Moreno, Patricio Barria, Rolando Aguilar, José M. Azorín, Marcela Munera, Carlos A. Cifuentes

**Affiliations:** 1 Department of Biomedical Engineering, Colombian School of Engineering Julio Garavito, Bogotá 111166, Colombia; daniel.gomez-v@mail.escuelaing.edu.co (D.G.-V.); felipe.ballen@mail.escuelaing.edu.co (F.B.-M.); marcela.munera@escuelaing.edu.co (M.M.); 2 Department of Electrical Engineering, University of Magallanes, Punta Arenas 6210427, Chile; pbarria@rehabilitamos.org (P.B.); rolando.aguilar@umag.cl (R.A.); 3 Club de Leones Cruz del Sur Rehabilitation Center, Punta Arenas 6210133, Chile; 4 Brain-Machine Interface Systems Lab, Systems Engineering and Automation Department, Miguel Hernández University of Elche UMH, 03202 Elche, Spain; jm.azorin@umh.es

**Keywords:** Powered Ankle-Foot Orthosis (PAFO), overground gait, ankle exoskeleton, biomechanical analysis, Gait Deviation Index (GDI), Movement Analysis Profile (MAP), Gait Profile Score (GPS)

## Abstract

Robotic devices can provide physical assistance to people who have suffered neurological impairments such as stroke. Neurological disorders related to this condition induce abnormal gait patterns, which impede the independence to execute different Activities of Daily Living (ADLs). From the fundamental role of the ankle in walking, Powered Ankle-Foot Orthoses (PAFOs) have been developed to enhance the users’ gait patterns, and hence their quality of life. Ten patients who suffered a stroke used the actuation system of the T-FLEX exoskeleton triggered by an inertial sensor on the foot tip. The VICONmotion capture system recorded the users’ kinematics for unassisted and assisted gait modalities. Biomechanical analysis and usability assessment measured the performance of the system actuation for the participants in overground walking. The biomechanical assessment exhibited changes in the lower joints’ range of motion for 70% of the subjects. Moreover, the ankle kinematics showed a correlation with the variation of other movements analyzed. This variation had positive effects on 70% of the participants in at least one joint. The Gait Deviation Index (GDI) presented significant changes for 30% of the paretic limbs and 40% of the non-paretic, where the tendency was to decrease. The spatiotemporal parameters did not show significant variations between modalities, although users’ cadence had a decrease of 70% of the volunteers. Lastly, the satisfaction with the device was positive, the comfort being the most user-selected aspect. This article presents the assessment of the T-FLEX actuation system in people who suffered a stroke. Biomechanical results show improvement in the ankle kinematics and variations in the other joints. In general terms, GDI does not exhibit significant increases, and the Movement Analysis Profile (MAP) registers alterations for the assisted gait with the device. Future works should focus on assessing the full T-FLEX orthosis in a larger sample of patients, including a stage of training.

## 1. Introduction

Stroke is the main cause of disability and the second leading cause of death worldwide [1]. People who suffered a stroke can be affected by after-effects such as hemiparesis, hemiplegia, communication disorders, cognitive deficits, or visual loss (i.e., partial or complete) [2]. Specifically, hemiparesis, which consists of weakness in one side of the body, is one of the most common neurological conditions. Another frequent consequence is spasticity, which causes increased muscle tone on account of the imbalanced signals of the central nervous system [3].

Specifically, people who suffered a stroke can exhibit dysfunctions such as foot drop. This pathology affects the ankle-foot complex’s control, this joint group being fundamental in human gait [4]. Specifically, foot drop is a neuromuscular disorder that impairs the ability of patients to move the foot along the sagittal plane [5,6]. Therefore, people with this condition present limitations such as low walking speed, altered gait pattern, and an increased risk of falling [6,7].

Likewise, the altered gait pattern results in high metabolic costs mainly related to compensatory movements in the non-affected joints (e.g., trunk bending, hip circumduction, or excessive flexo-extension in the hip and knee joints) [6,7]. These movements intend to counteract the loss of motor functions and achieve functional skills in a pathological gait, although increasing the risk of permanent damages in the locomotor system [4,8,9]. Moreover, those pathological conditions interfere in the proper execution of different Activities of Daily Living (ADLs) (e.g., sit-to-stand, walking, or going upstairs or downstairs), limiting the patients’ independence and their quality of life [10].

Conventional physical therapy in a rehabilitation scenario has been widely used to overcome the conditions mentioned above [2,11]. Mainly, therapy helps to improve neurological recovery and patient’s motor functions [11]. Hence, rehabilitation processes include training in both task-specific and context-specific, particularly in the early stages after injury [2]. This way, patients improve motor skills to accomplish multiple activities such as bed mobility, body motions to execute ADLs, and patient-environment interaction using a wheelchair [12].

According to the importance of walking and its influence on people’s quality of life, rehabilitation programs are also focused on this capability’s recovery. Therefore, rehabilitation processes implement several intervention methods (e.g., classical gait rehabilitation techniques, functional electrical stimulation, gait support orthoses, robotic devices, and brain-computer interfaces) [13]. In terms of ankle rehabilitation, the methods look to enhance patient parameters such as, balance, motor control, and foot clearance, among others [14].

Considering the techniques previously mentioned, passive Ankle-Foot Orthoses (AFOs) are the most common solution for patients with ankle impairments [15]. This device is a mechanical structure used to correct ankle-foot deformities, lock the ankle for improving stability, and provide a certain degree of independence for walking [16]. Nevertheless, passive AFOs do not assist the ankle movements, and hence, patients need to compensate the dysfunction through the hip and the knee joints. Therefore, aspects such as the abnormal gait pattern and the risk of permanent damages to the locomotor system remain for the patient [17].

In this context, rehabilitation programs are motivating the development of Powered Ankle-Foot Orthoses (PAFOs) [18], based on the promising results of robotics applied to physical therapy [19,20,21]. This way, PAFOs could enhance the patients’ gait patterns, retraining the affected functions and achieving an increased motor rehabilitation. Likewise, novel control strategies and different actuation principles are being applied in those robotic orthoses to improve both human-robot interaction and patients’ recovery capacity [18,22].

T-FLEX [23] is a wearable and portable PAFO for rehabilitation and assistance, which can be manually adjustable and suitable for both limbs. This device has two servo motors placed on the anterior and posterior parts of the shank. T-FLEX integrates an inertial sensor and a statistical algorithm to estimate the user gait phase in real time [24]. Hence, the device assists in dorsi-plantarflexion movements during the gait phase transitions and reduces the resultant torque on the ankle during the stance phase.

This PAFO is part of a small group of exoskeletons with compliant actuators and soft structures referred to as fully compliant exoskeletons [18]. Moreover, considering the mechanical design, T-FLEX has a high potential for applications in portable scenarios [23]. Likewise, in the rehabilitation context, T-FLEX reports promising results for a stationary therapy, registering a recovery of the motor capabilities of patients with stroke who exhibit spasticity [25].

From the encouraging results in therapy and the potential application in gait assistance, this work presents the assessment for the first use of the T-FLEX’s actuation system in overground gait. The main goal of this study is aimed at measuring the changes in kinematic and spatial-temporal parameters between the two conditions proposed (i.e., unassisted and assisted modalities). Additionally, it also intends to determine the level of satisfaction of the user with the device, in aspects such as dimensions, weight, safety, and comfort.

## 2. Materials and Methods

### 2.1. Powered Ankle-Foot Orthosis

**Figure 1:**
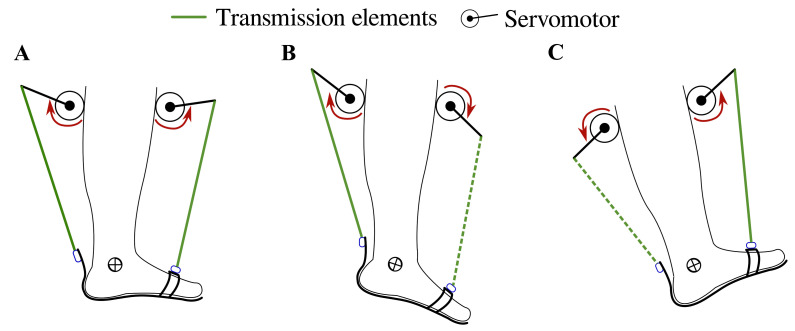
T-FLEX’s actuation system states for gait assistance. The red arrows indicate the actuator direction of rotation to assist (**A**) stance phase, (**B**) propulsion during toe-off, (**C**) and foot clearance in swing and heel strike phases. The segmented and continuous lines refer to the transmission elements’ participation in each movement, i.e., in plantarflexion, only the posterior element works, and in dorsiflexion only the frontal element is transmitted.

T-FLEX is a portable and wearable Powered Ankle-Foot Orthosis (PAFO) designed from bioinspired concepts to assist and rehabilitate people with ankle dysfunctions [23,25]. T-FLEX is composed of two servomotors, MX106 (Dynamixel, Korea), placed on the anterior and posterior part of the user’s shank. The actuators emulate the functionality of the muscles (i.e., agonist and antagonist movements) to provide the dorsi-plantarflexion movements on the ankle (see Figure 1).

Furthermore, T-FLEX integrates an inertial sensor BNO055 (Bosch, Germany) with a sample rate of 100 Hz on the foot tip. In addition to that, a statistical algorithm, based on the hidden Markov model method and adapted for real-time applications (i.e., changing the standard Viterbi procedure to forward-only Viterbi), estimates the user’s gait phases [24]. Thus, the algorithm compares online the user’s angular velocity and acceleration, measured on the sagittal plane, with a model trained previously. The machine learning models correspond to signals of patients with ankle dysfunctions acquired in a previous study [24].

The control strategy intends to assist the dorsi-plantarflexion on the ankle according to the gait phase detected by the algorithm (i.e., heel strike, flat foot, toe-off, and swing phase). For the stance phase, the actuators turn in the same direction to provide stability and balance to the user (see Figure 1A), causing a net torque close to 0 Nm on the ankle. On the other hand, the motors operate in opposite directions to provide both torque propulsion on the heel strike and foot clearance during the swing (see Figure 1B,C). The control system and the gait phase detector run under an ROS (Robot Operating System) architecture in a Raspberry Pi 3.

Taking into account the purpose of this study, a passive orthotic structure (Han River, Beijing, China) integrated the actuation and control systems of T-FLEX, following the recommendations of the Club de Leones Cruz del Sur Rehabilitation Center’s Ethics Committee. The structure is composed of an insole adapted with Velcro strips and a mechanical system to limit the ankle movements on the sagittal plane. Moreover, it has an adjustable mechanism to increase the distance between the motors and the insole. The passive orthosis coupled with the T-FLEX’s actuation system has a total weight of 2.8 kg (i.e., 1.9 kg for the structure placed on the limb and 0.9 kg for electronic components located on the hip), remaining within the reported range of ankle exoskeletons [18]

**Figure 2:**
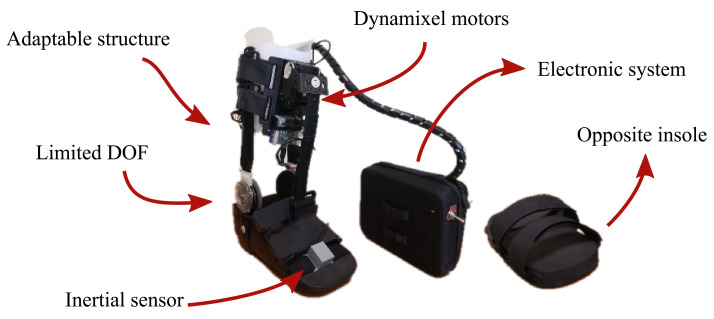
The actuation system of the T-FLEX exoskeleton that was implemented on the passive orthotic structure. The insole of the left part is added to the non-paretic limb to compensate for the effect due to the device’s use.

On the other hand, this protocol also included an opposite insole placed on the healthy limb to compensate for wearing the device. Figure 2 shows the adapted structure and the opposite insole used in this experimental validation. On the whole, this study solely assessed the actuation system of T-FLEX; hence, the passive structure described was included to ensure the fixation of the actuators to the user and guarantee a proper force transmission during the gait assistance.

### 2.2. Participants

This study enrolled 10 participants (58±4.5 years old) diagnosed with hemiparesis due to a cerebrovascular accident (i.e., eight males and two females). They were active patients who performed therapy processes in a rehabilitation center. Table 1 summarizes the clinical information of the patients who accomplished this study. On the other hand, the volunteers were selected according to the inclusion and exclusion criteria described below:

- Inclusion criteria: People who suffered a stroke before six months of executing this protocol were eligible. The volunteers must present hemiparesis on one side of the body with some ankle dysfunction. Moreover, they must have partial independence for walking without external devices and the ability to follow instructions.
- Exclusion criteria: Candidates with skin alterations in the lower limbs, a high level of spasticity (i.e., Level 4 on the Ashworth scale), and pain of the musculoskeletal system that impedes the use of the device were not included in this study, as well as patients who suffered from weakening diseases, for instance, cancer. Moreover, people with a previous history or suspected of seizures were also not selected.

### 2.3. Experimental Setup

**Figure 3:**
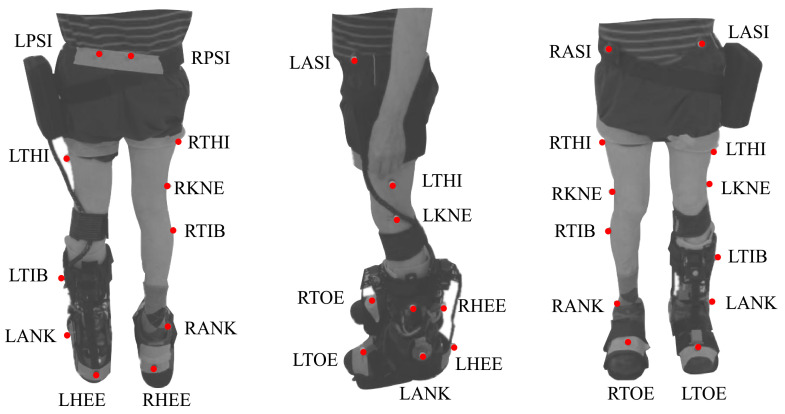
Biomechanical setup model used in the study for each participant based on the plug-in gait marker model. The red points on the patient represent the markers and the biological landmarks for the VICON acquisition system. This model involves markers in the Right and Left Posterior Iliac Spines (RPSI and LPSI), Right and Left Anterior Superior Iliac Spines (LASI and RASI), Right and Left Thighs (RTHI and LTHI), Right and Left Knees (RKNE and LKNE), Right and Left Tibias (RTIB and LTIB), Right and Left Ankles (RANK and LANK), Right and Left Toes (RTOE and LTOE), and Right and Left Heels (RHEE and LHEE).

This protocol included two modes (i.e., baseline and assisted gait) to analyze the effects of the T-FLEX’s actuation system. For both modes, participants were instrumented with 25 markers under a Plug-in Gait marker model [26]. Besides, trials were executed on a straight path of 6 meters, where ten cameras, VICON (Oxford Metrics, Oxford, UK), were distributed to acquire the user kinematics. Figure 3 shows the distribution and the biological landmarks of the markers over a volunteer of this study.

On the other hand, for the assisted gait mode, the participants used the actuation system of T-FLEX adapted to a mechanical orthotic structure on their paretic side (see Figure 3). Likewise, the gait phase detector employed an inertial sensor placed on the same limb’s foot tip (see Figure 2). The actuators was configured to the maximum velocity (55 rpm for the no-load state) to assist the ankle movements (i.e., dorsiflexion and plantarflexion) along the path. On the other foot, the volunteers also used a similar insole to balance the effect due to the device’s height (see Figure 2).

### 2.4. Experimental Procedure

From the two modalities proposed for this study (i.e., baseline and assisted gait), each participant accomplished multiple 6 Meter Tests (6MTs) overground during the same session. On the other hand, a VICON motion capture system recorded the patients’ kinematics using the markers’ distribution shown in Figure 3. Likewise, a physical therapist followed the participant during the trials to guarantee the patients’ safety in case of an unexpected event.

The unassisted gait (baseline) consisted of walking without wearing the device. Thus, each participant walked ten times along a straight path of 6 meters. This way, trial data were used as the reference for the kinematic analysis. In the second modality, the device assisted user gait in the same path ten times, according to the control scheme shown in Figure 1. Therefore, a calibration stage was executed to record the user’s Range Of Motion (ROM) through T-FLEX’s actuators.

Trials were executed continuously in both modalities with a resting time between conditions to adjust the participant setup. Moreover, the modalities were alternated during the experiment to objectify the usability assessment. On the other hand, the acquired trajectories were analyzed and compared to identify the curves with the highest intra-test consistency. Thus, those selected curves were used for the biomechanical analysis presented in the previous section.

The experimental procedure was executed by members from the Movement Analysis Laboratory of the Rehabilitation Corporation Club de Leones Cruz del Sur (Punta Arenas, Chile).

### 2.5. Biomechanical Analysis

Nexus software (Oxford Metrics, Oxford, UK) was used to track the trial data, and Polygon software (Oxford Metrics, Oxford, UK) provided the kinematic outcomes of each user. In this sense, a plug-in gait model was used to calculate the spatial-temporal parameters such as the percentage of the stance phase, step width, step length, cadence, and walking speed. Furthermore, this model allowed estimating the three-dimensional movements on the lower limb joints (i.e., flexo-extension, abd-adduction, and internal-external rotation).

On the other hand, the Gait Deviation Index (GDI), which synthesizes all the variables of the kinematic examination in a single general result, was estimated for each participant’s leg [27]. The obtained value represents a percentage of global normality, compared to a kinematic reference of people without pathology or mobility alterations. Therefore, values greater than 90% indicate a non-pathological gait pattern in the limb. This index allows identifying changes in joint kinematics (i.e., variations above 10%) for several scenarios [28]. The procedure to estimate GDI was deeply detailed in [27] and is available to be used in a public dataset at https://doi.org/10.6084/m9.figshare.12576965.v1 (accessed on 27 June 2020).

Other measures used to detail the kinematic performance were the Movement Analysis Profile (MAP) and the Gait Profile Score (GPS) [29]. The MAP describes the magnitude of the deviation on the lower limb joints across the gait cycle, and the GPS compiles and averages those joints’ scores. The methodology to calculate the GPS and MAP was detailed in [29] and is available for this study in a public dataset at https://doi.org/10.6084/m9.figshare.12576965.v1 (accessed on 27 June 2020).

### 2.6. Usability Assessment

Ergonomics and comfort are some of the most relevant aspects of user-machine interaction [30]. For this study, the user perception assessed this interaction employing a Quebec User Evaluation of Satisfaction with assistive Technology (QUEST) test. The original survey was composed of 27 questions related to participants’ satisfaction concerning the robotic device [31]. This study included 13 of those questions adapted to a Spanish version, which were selected for their suitability in this protocol.

### 2.7. Statistical Analysis

This study analyzes the effect on the biomechanical and spatial-temporal parameters of the device during its first use on patients with stroke. For this purpose, initially, a Shapiro–Wilk test verified the normal distribution of the data. This way, the data segmented by gait phases (i.e., stance phase and swing phase) were averaged for each subject.

Subsequently, Student’s *t*-tests assessed the statistical changes (*p* < 0.05) between the baseline and assisted gait with the T-FLEX’s actuation system for both gait phases. This part included inter-subject (between-participant) and intra-subject (within-participant) tests to analyze the first use effects. Thus, it allowed measuring aspects such as user performance, adaptability to the device, and the influence of actuating the ankle joint.

On the other hand, the spatial-temporal analysis was also performed by the Student’s *t*-test between the two conditions for intra-subject tests. The software used for the tests was MS Excel with statistical analysis tools.

## 3. Results

### 3.1. Kinematics

In this study, the kinematic results of the users were divided into two main groups: (1) the behavior of the ankle kinematics and (2) the Range Of Motion (ROM) of the lower limb joints. As an initial approach, the ankle kinematics showed no significant changes (*p* > 0.05) for the two groups (i.e., unassisted and assisted), including the complete sample of participants through a Student’s *t*-test. Nevertheless, diverse aspects stated in the following section could explain those results. Therefore, this part presents the results individually for each participant.

**Figure 4:**
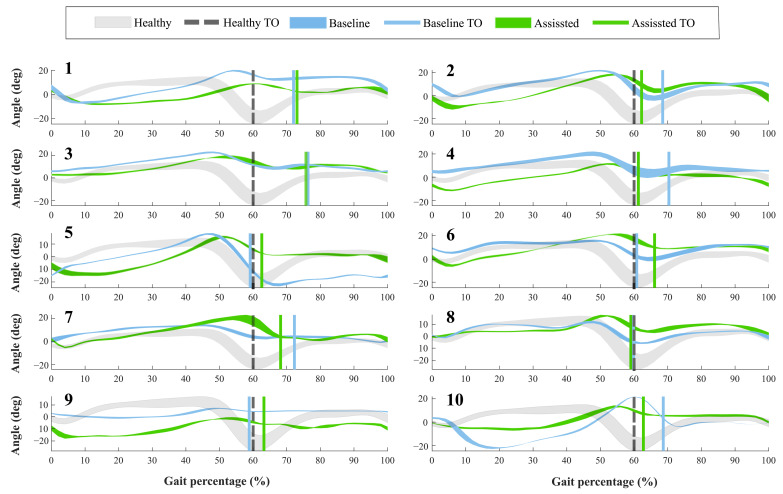
Volunteers’ ankle kinematics during the gait cycle. Numbers on the left part represent the assessed participants. The green curve indicates the assisted gait condition. On the other hand, the blue curve refers to the natural gait pattern (i.e., baseline condition). The gray curve shows a healthy gait pattern obtained from a database of people with no pathological gait available in Figshare at a public repository (https://doi.org/10.6084/m9.figshare.12576965.v1 (accessed on 27 June 2020)). Finally, the vertical lines describe the Toe-Off event (TO) for each of these conditions.

For the first group, Figure 4 shows the ankle kinematics during a gait cycle for a healthy pattern and the results of each volunteer. This cycle comprises phases between each heel-strike event. Moreover, the vertical line included in the figure highlights the toe-off state for both modalities assessed (i.e., baseline and assisted gait) and the healthy ankle pattern.

Concerning the Toe-Off phase (TO), forty-percent of the participants showed differences of more than 5% in the occurrence of this event, during the assisted gait (see Figure 4). Likewise, thirty percent of the subjects brought this event to the estimated percentage in a healthy pattern. The other volunteers did not show changes in this aspect. On the other hand, the ankle angle shape had variations when the participants wore the device. Specifically, Subject 5 registered an increase of 15 degrees in the dorsiflexion movement during the swing phase. However, Participants 1 and 9 reduced this movement at 10 degrees, although this reduction was within the healthy range.

For the other group, Table 2 summarizes the ROM for the Ankle dorsi-plantarflexion (A-F), Knee Flexo-extension (K-F), Hip Flexo-extension (H-F), and Hip Abduction-Adduction (H-A) in both modalities. The second part of the table shows the percentage variation of the joints when the participant used the T-FLEX orthosis. Positive values in this variation indicate an increase in the joint’s ROM, and by contrast, negative values represent a decrease in this parameter. For this part, the highlighted values represent increases greater than 10% on the joint concerning the baseline state.

From the variation table, seventy percent of the volunteers exhibited significant changes in the paretic ankle ROM using the device, whether increases or decreases. Likewise, the changes in the ROM for the paretic ankle also tended to vary for the non-paretic joint. On the other hand, the number of altered joints was directly proportional to the change presented on the ankle, where values of the paretic ankle ROM with variation above 50% registered changes in at least half of the analyzed joints. In general, the changes did not show a common tendency in terms of increases or decreases. Furthermore, the larger values corresponded to changes on the dorsi-plantarflexion (A-F), although Subjects 4, 5, and 7 showed the Hip Adduction (H-A) value as the maximum variation.

**Figure 5:**
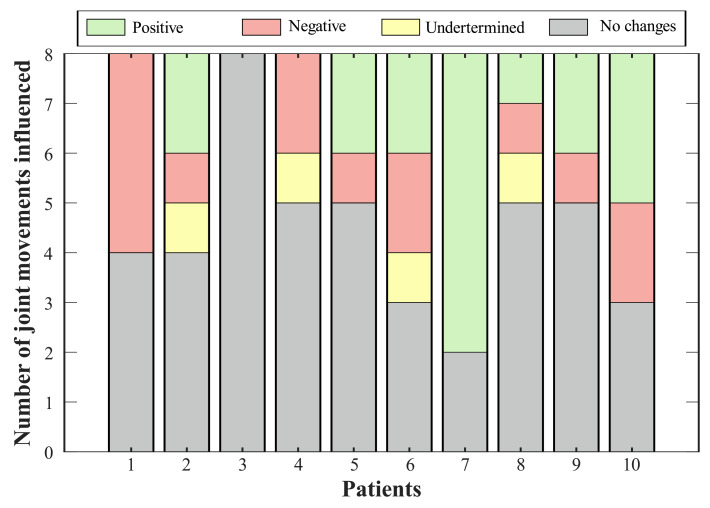
Effect of T-FLEX scenario on the joints’ range of motion. Positive changes (green bar) refer to variations that approach the value of a healthy pattern. Negative changes (red bar) comprehend joints where the ROM departs from the normal gait. Undetermined conditions (yellow bar) integrate magnitudes that exhibit variation, but they do not generate an improvement or an impairment. Lastly, no change states (gray bar) include percentages of less than 10%.

According to the variations on the ROM of the lower limb joints (see Table 2), it is essential to determine whether this change represents a positive or negative effect in the joint of the participant (see Figure 5). The ROM obtained was compared with the mean value in a healthy gait [32]. In this context, sixty percent of the volunteers showed improvement in the dorsi-plantarflexion (A-F) using the device. Among these, Subjects 2, 5, and 7 achieved values whose errors, regarding the ROM in healthy people, were less than 2%. This way, positive changes in the paretic ankle joint improved the ranges for the non-paretic joints, especially in the ankle joint. For 30% of the participants, the variations in the dorsi-plantarflexion (A-F) did not represent significant improvements, and additionally, one volunteer exhibited a negative effect in this ROM related to a reduction of 33% in its value.

Thus, Figure 5 summarizes the consequences of using the T-FLEX system actuation on the analyzed joints for each participant. The positive effects indicate improvement in the ROM of the corresponding joint, this value approaching healthy ranges. Negative impacts show a pattern disruption, and hence a distancing of the movement from a healthy pattern. Undetermined conditions grouped changes where, although the variation was significant (i.e., above 10%), this value did not improve or impair the ROM. Lastly, the no-changes group integrates the differences between both scenarios of less than 10%.

Bearing in mind the classification of variations for each subject (see Figure 5), seventy percent of the volunteers showed a positive effect on at least one joint, where the paretic ankle was the more prevalent (i.e., for six participants). The exhibited negative impacts were mainly related to reductions in the hip, although Participant 1 registered a decrease in the dorsi-plantarflexion, both knees, and the non-paretic hip adduction-abduction. On the other hand, two joints reflected increases (i.e., knee flexo-extension and hip adduction-abduction for the non-paretic limb) that did not represent a risk for the participant.

On the other hand, Table 3 contains the Gait Deviation Index (GDI) for each participant. The GDI showed a significant difference for 30% of the participants’ paretic limbs, wherein 20% manifested a reduction below 14% and one volunteer registered an increase of 14%. For the non-paretic, forty percent of the participants exhibited a decrease by less than 30% for this index. Reduction in GDI was related to a higher difference between the participant kinematics and a healthy pattern. In contrast, an improvement in the gait kinematics caused an increase of this index. The mean value of GDI for the assessed group did not present a significant difference between the scenarios, and both limbs remained in the un healthy range because the GDI percentage was less than 90% (see Table 4).

**Figure 6:**
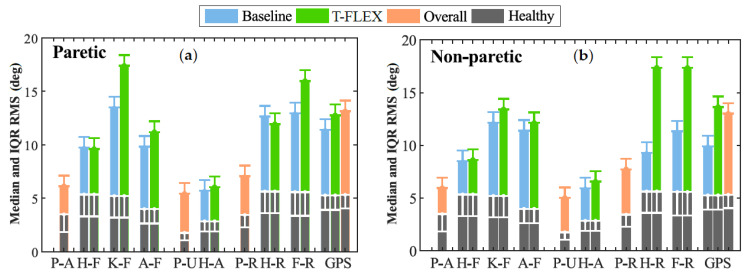
Movement analysis profile. Each column represents one of the kinematic variables such as P-A (Pelvis Anterior-posterior), H-F (Hip Flexion-extension), K-F (Knee Flexion-extension), A-F (Ankle dorsi-plantarflexion), P-U (Pelvic Up-down), H-A (Hip Abduction-Adduction), P-R (Pelvic Rotation), F-R (Foot Rotation), and GPS (Gait Profile Score). The height of the bar indicates the median and IQR RMS value during the trial. The gray columns at the bottom denote the mean values for a healthy gait pattern obtained from [29]. Those values are used as the reference to compare the unassisted condition (blue bars) and assisted gait (green columns).

Lastly, Figure 6 illustrates the Movement Analysis Profile (MAP) for the paretic (Figure 6a) and non-paretic (Figure 6b) limbs between baseline and assisted gait. The most affected movements on the joints were (1) the Foot Rotation (F-R) in both limbs, (2) the Knee Flexo-extension (K-F) for the paretic side, and (3) the hip rotation in the non-paretic.

The ankle dorsi-plantarflexion did not show significant changes in both the paretic and non-paretic for the assessed modalities. On the other hand, the Gait Profile Score (GPS) significantly increased its value between unassisted and assisted conditions for the non-paretic limb. This change moved the index away from the value of healthy people; hence, the gait was negatively affected when the user wore the device and the adapted insole. Nevertheless, this value did not exhibit significant changes for the paretic side.

### 3.2. Spatial-Temporal Parameters

Considering the variation in the ROM presented above, the second part of this work analyzes the changes of the spatial-temporal values in the proposed modalities. In this sense, Table 5 shows the variation percentages of the participants’ parameters that were determined in this study. These parameters included mean values for the paretic and non-paretic limbs in aspects such as the duration of the stance phase, step length, and step width. Likewise, variations in walking speed, stride length, and cadence were also estimated.

In general terms, the spatial-temporal parameters did not show significant changes using the T-FLEX actuation system to either of the participants’ limbs. Nevertheless, the cadence exhibited a reduction in 70% of the volunteers. This parameter registered decreases below 24% of the baseline state, although Subject 8 presented an increase in the cadence of 20% for the assisted gait.

On the other hand, Table 4 contains the mean values for the assessed group parameters. Specifically, the group showed a decrease in the cadence value of 14% (i.e., from 99 to 85 steps per minute) when the participants used the T-FLEX’s actuation system. Likewise, the walking speed also decreased 0.1 m/s, registering values of 0.8 for the baseline and 0.7 m/s in assisted gait.

### 3.3. Usability Assessment

This part describes the device performance in terms of user-machine interaction and the perception of the participants with assistive technology. Firstly, no patient exhibited issues (i.e., affectations in the locomotor system, pressure points, skin injuries, or falls) during and after wearing the device.

**Figure 7:**
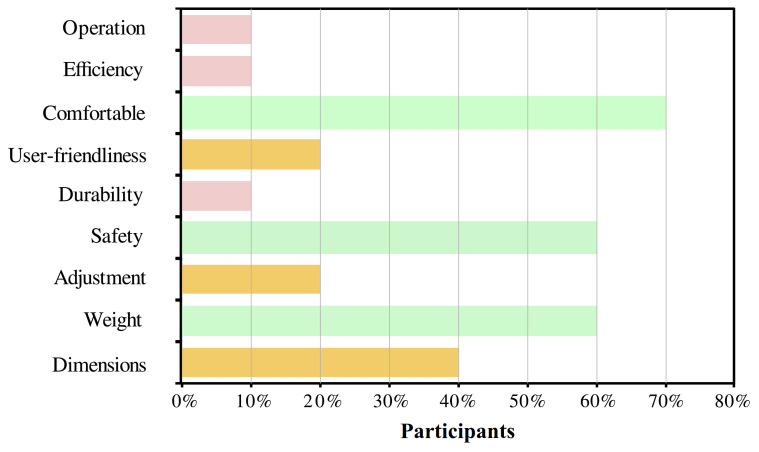
Results of the usability assessment through the Quebec User Evaluation of Satisfaction with assistive Technology (QUEST) test. The percentage of each topic refers to the number of participants who considered that characteristic as relevant.

For the users’ perspective, Figure 7 shows the relevant aspects selected by the participants through the QUEST survey. The most selected parameter was the device’s comfort with 70% of recurrence. Other important aspects for the users were safety, weight, and dimensions. Finally, the level of satisfaction of the user was between satisfied and very satisfied in 60% and 40% of the users, respectively.

### 3.4. Statistical Analysis

To understand the participants’ effects on the gait cycle, the statistical analysis aimed to identify differences between assisted and baseline conditions. In terms of the ankle kinematics, the results revealed statistically significant changes for 70% of the subjects in at least one gait phase for the angle. Specifically, this joint showed statistical differences in the stance and swing phase for 60% and 70% of the participants, respectively (see Table 6). Moreover, forty percent of them exhibited variations in the entire gait cycle.

In the spatial-temporal context, the parameters showed a statistically significant decrease in the cadence (*p* = 0.0002) and speed (*p* = 0.03) concerning the assisted gait. The parameters of long stride, step length, step width, and stance phase did not show statistical differences.

## 4. Discussion

The results shown in the previous section present the effects on the lower limb joints for the assisted gait with the actuation system of T-FLEX. For that, the kinematics presented the results for the participants individually. This analysis allowed determining aspects such as the participant performance during the trial, adaptability to the device, improvement in the ankle kinematics, and the consequences on the other planes of motion. On the other hand, an inter-subject (i.e., between-subjects) analysis did not evidence significant changes comparing unassisted and assisted gait. However, those results could be affected by the poor performance exhibited by some participants. Specifically, this performance can be related to the lack of a training stage with the device and the non-customized model used by the gait phases algorithm during the assisted gait. Thus, aspects such as changing the experimental protocol to include the training stage and customizing a machine learning model for each patient could improve the user’s performance and adaptability to the device.

In this context, the ankle kinematics described the device’s influence in this joint for each user (see Figure 4). From the significant changes found in the gait cycle, the T-FLEX’s actuation system positively impacted the dorsiflexion movement in three patients. This way, the device improved the ankle joint kinematics, providing foot clearance during the swing phase. Therefore, the device reduced the risk of falls and injuries [33]. For the stance phase, subjects exhibited reductions in the ankle joint angles for the assisted gait. Thus, this behavior could be interpreted as a better fixation of the foot to the ground that would provide stability. Other changes related to the limited assistance of the dorsi-plantarflexion movements could be associated with the user-device synchronization (i.e., gait phases algorithm) and the calibration stage carried out manually (i.e., ankle ROM values recorded by the actuators). However, the limited device’s assistance did not represent a risk for the users’ stability because the T-FLEX’s actuation system does not restrict the ankle movements. On the other hand, taking into account the first 10% of the gait cycle, sixty percent of the volunteers exhibited a kinematic behavior similar to the shape of the healthy pattern. Likewise, assisted gait also showed a smoother transition between phases, ensuring a suitable joint control to provide stability and safety.

In general terms, the kinematic results during the first use of the T-FLEX actuation system showed improvements in some participants (i.e., increased foot clearance and early push-off), which are similar to a robust PAFO based on pneumatic actuation [34]. Additionally, these results are comparable to devices controlled by a Force Sensitive Resistor (FSR) for gait detection [35,36], which is the most common detection strategy used in wearable robotic orthoses. Nevertheless, those previous studies enrolled a smaller sample of subjects, reducing the probability of poor performance in the participants. Lastly, the ankle kinematics results also tended toward the outcomes of another study that included a training stage [37], unlike this protocol.

Gait performance can also be analyzed through the other joints of both paretic and non-paretic sides [38]. Usually, this assessment includes at least the knee and ankle joints, where the results commonly exhibit improvement of the kinematics [35]. This study showed proper adjustment of the ankle’s ROM to avoid foot drop, through the mechanical structure that limits the sagittal plane, as well as the T-FLEX actuation system. In the hip context, the adduction-abduction (H-A) decreased in 70% of the participants for the non-paretic limb. This reduction is a result of the restriction and actuation on the paretic ankle. In contrast to the non-paretic, the other side presented disruptions in 40% of the subjects related to reductions in the ROM value.

In particular, Subject 7 showed high performance in the estimated ROM for both sides. The positive effects were in 75% of the analyzed joints with the best improvement in the dorsi-plantarflexion (A-F) for the non-paretic limb. This outcome could not be associated with the user’s spasticity level because Subjects 2 and 8 have clinical conditions comparable to this participant, but they did not exhibit similar performance. Hence, it could relate to external variables such as correct synchronism of the device and the appropriate actuation performance.

Spatial-temporal parameters allow measuring the device’s effects on the user [22,38]. Mainly, orthotic devices should improve the subjects’ parameters to enhance their mobility in the execution of ADLs [39]. The first use of T-FLEX showed a decrease in cadence. This reduction is related to both the training stage (not included in this study) and the restricted structure on the ankle. Therefore, the inclusion of training stages could be imperative to improve the obtained results [40].

On the other hand, Table 3 shows the GDI and the variation according to each scenario. Regarding the baseline, most of the participants decreased their GDI, although only 30% of the limbs registered a reduction above 10%. Several factors can explain the decrease in this index. The first factor is related to the MAP information (see Figure 3), where the foot rotation represents one of the most significant movements with affectations. This alteration is due to the mechanical structure coupled to the T-FLEX actuation system. Moreover, the restriction on the ankle triggers a disruption in the other joints’ patterns [6,7,8], which could induce a decline in this index. The second factor comprehends the performance of the actuation system in aspects such as response time to position set-points, processor speed for running the detection algorithm, and the manual calibration stage that recorded the maximum flexo-extension angles of the user. On the other hand, multiple studies have presented GDI analysis for children with cerebral palsy using a passive orthotic device [41,42,43]. However, in studies that involve patients with stroke using PAFO, this index was not shown.

Finally, in the MAP context (see Figure 6a), assisted gait with T-FLEX affected several movements on the paretic joints, e.g., Knee flexo-Extension (K-F), dorsi-plantarflexion (A-F), and Foot Rotation (F-R). Nevertheless, as mentioned previously, the changes could lead to the passive structure adapting and the mechanical restriction on the ankle. This way, the structure alters the natural gait pattern and induces compensatory motions on the other joints [6,7,8], although the lack of training could also cause this wrong pattern. For the non-paretic side (see Figure 6b), the main affectations were the Hip Rotation (H-R) and Foot Rotation (F-R) movements, which could be related to the device’s weight compensation. As for the GDI, different studies used the GPS to analyze the effects on people with cerebral palsy [44,45]. Although, in protocols that include patients with stroke in assisted gait with PAFO, the score was not reported.

In summary, this experiment exhibited no changes and positive and negative effects in the participants’ kinematic parameters when they walked with the T-FLEX’s actuation system. Multiple reasons exposed in this section could respond to the low device performance in several patients. However, although the use of the T-FLEX’s actuation system in gait assistance is not conclusive, the obtained results evidenced the device’s advantages in avoiding foot dragging during assistive applications. These results are mainly related to improvements in patients with high adaptability in aspects such as the toe-off event and ankle kinematics.

Likewise, it is essential to remark that this study was executed in a passive orthotic structure that is not part of the T-FLEX exoskeleton. Therefore, the kinematic parameters could be also affected by the ankle motion’s restriction (i.e., eversion-inversion and adduction-abduction) and the structure’s weight. Specifically, considering the total device’s weight, increased joint motions (e.g., knee and hip flexion) could be exhibited to guarantee a proper swing phase. This way, the gait pattern and spatial-temporal aspects might evidence benefits related to the ankle actuation, but also disadvantages due to the increased movements.

Nevertheless, despite these effects, patients who presented proper synchronization and adequate manual calibration evidenced improvements in their lower limb kinematics (i.e., closing the kinematics to healthy ranges), which were related to the device’s suitable assistance. This way, the T-FLEX exoskeleton could also exhibit potential use in gait rehabilitation for assistive scenarios, based on compensatory movements’ reduction to improve the pathological gait pattern.

## 5. Conclusions

This work presents an assessment of the T-FLEX actuation system during its first use. For that, ten patients who suffered a stroke wore the device in overground walking. In the inter-subject analysis context, the biomechanical analysis showed improvements for some patients in dorsiflexion to avoid foot falland control of the ankle in the phase transition. Moreover, the other joints exhibited positive and negative changes related to the actuation on the paretic ankle with T-FLEX. For the intra-subject analysis, the results showed no significant differences between baseline and assisted gait. This value could be related to the limited assistance performed by the T-FLEX’s actuation system (i.e., gait phase detector, manual calibration, and passive orthotic structure’s effects) with several participants.

Spatial-temporal parameters did not present significant changes, although the cadence decreased for the assisted gait. Lastly, the GPS and GDI measured the kinematic behavior for each participant in both modalities. Those parameters did not evidence significant improvements between subjects and a healthy pattern, and they also determined the main joints affected by the device.

Lastly, this study found that the T-FLEX’s actuation system could not be intuitive for use in the first trial with patients who exhibit a stroke. Therefore, a training stage could be necessary to familiarize the user with the device (i.e., in aspects such as the device’s dimensions and weight) and synchronize the system properly during gait assistance scenarios.

Future works should focus on the assessment of the full T-FLEX orthosis in a more extensive sample of patients with stroke. Additionally, the device’s calibration stage and the performance of actuation should be optimized to improve the presented results. Further studies will also aim to complete gait analysis after a training stage, which will allow measuring the biomechanical and kinetic effects on the users.

## Figures and Tables

**Table 1:** Subjects’ anthropometric measurements and clinical information.

Subject	1	2	3	4	5	6	7	8	9	10	$\bar{x}\pm \mathrm{sd}$
Gender	Male	Female	Female	Male	Male	Male	Male	Male	Male	Male	-
Age (years)	54	52	59	54	61	61	66	60	60	53	58 ± 4
Weight (Kg)	80	91	95	87	96	62	67	73	69	84	80 ± 12
Height (cm)	170	165	167	175	168	160	170	166	165	176	168 ± 5
Left leg lenght (cm)	90	87	88	91	88	83	88	88	85	92	88 ± 3
Right leg lenght (cm)	90	87	88	91	88	83	88	88	85	93	88 ± 3
Stroke diagnosis	Ischemic	Ischemic	Ischemic	Ischemic	Ischemic	Ischemic	Ischemic	Ischemic	Ischemic	Ischemic	-
Time from injury (years)	2	7	5	1	7	2	4	4	4	5	-
Paretic side	Right	Right	Right	Left	Left	Right	Right	Left	Left	Left	-
Ashworth scale	1	1	2	1	3	1	2	2	1	3	-

**Table 2:** Range of motion on the participants’ lower limb joints in the proposed scenarios (i.e., baseline and T-FLEX). The analyzed movements comprise Flexo-extension on the Ankle, Knee, and Hip joints (A-F, K-F, and H-F, respectively). Moreover, it also includes the Adduction-Abduction on the Hip (H-A). Values in parenthesis represent the standard deviation. The lower part shows the percentage of variation on the joints when the participant used the T-FLEX’s actuation system. The positive values refer to increases in this value in contrast with the negative values, which indicate decreases. The highlighted values indicate significant joint changes greater than 10% for both increases (green) and decreases (red).

Subjects			1	2	3	4	5	6	7	8	9	10
Baseline	Paretic	A-F	27.4 (1.4)	26.1 (1.3)	17.9(1.3)	22.1 (0.9)	40.5 (2.1)	15.5 (2.2)	17.0 (1.1)	16.2 (0.5)	7.2 (0.8)	44.4 (1.2)
		K-F	62.4 (2.3)	51.7 (2.5)	29.6 (1.3)	70.9 (1.1)	46.6 (1.4)	64.2 (1.8)	18.4 (1.5)	49.4 (2.3)	31.1 (2.1)	40.1 (1.8)
		H-F	39.5 (2.6)	44.4 (1.7)	26.8 (2.5)	42.2 (1.3)	27.1 (0.3)	53.7 (3.7)	16.2 (3.6)	41.8 (0.9)	31.0 (2.5)	35.9 (1.4)
		H-A	7.5 (0.7)	13.9 (0.7)	12.1 (1.8)	11.8 (0.7)	8.1 (0.7)	10.2 (1.3)	10.1 (2.1)	8.4 (0.5)	8.6 (1.2)	11.2 (0.7)
	Non-paretic	A-F	16.2 (2.3)	35.0 (2.3)	21.3 (1.3)	20.5 (1.5)	23.3 (2.2)	18.1 (3.2)	19.6 (3.7)	16.8 (0.5)	20.2 (1.6)	38.7 (2.9)
		K-F	60.1 (1.7)	67.1 (2.4)	33.7 (1.6)	71.5 (0.9)	62.0 (0.8)	62.5 (1.8)	51.9 (0.6)	65.0 (0.9)	71.4 (2.0)	68.1 (1.1)
		H-F	39.3 (0.9)	48.2 (1.3)	38.7 (1.0)	46.1 (0.7)	39.3 (1.1)	48.7 (2.0)	39.4 (2.5)	54.9 (1.5)	49.5 (0.9)	52.1 (1.5)
		H-A	9.9 (1.8)	15.3 (1.8)	12.7 (0.5)	12.1 (1.4)	10.1 (1.0)	10.3 (1.4)	4.9 (1.2)	8.3 (1.0)	19.5 (1.4)	14.8 (2.0)
T-FLEX	Paretic	A-F	17.2 (0.8)	29.7 (2.5)	17.0 (3.5)	23.4 (1.4)	29.9 (1.3)	26.3 (2.5)	30.6 (3.8)	17.3 (0.9)	14.3 (2.78)	20.8 (2.0)
		K-F	50.6 (3.4)	61.2 (3.1)	31.7 (2.7)	65.1 (1.7)	41.3 (1.9)	53.1 (1.1)	20.3 (2.6)	51.2 (1.1)	29.3 (1.4)	39.4 (4.4)
		H-F	38.4 (0.6)	47.5 (2.5)	26.9 (1.8)	41.1 (0.9)	30.5 (0.9)	53.1 (3.0)	31.9 (4.8)	37.6 (1.2)	25.2 (2.8)	31.7 (2.5)
		H-A	7.7 (1.5)	14.8 (1.8)	11.7 (0.8)	8.7 (1.6)	14.1 (4.7)	6.5 (1.3)	8.9 (1.2)	7.2 (1.1)	9.8 (0.7)	8.8 (0.9)
	Non-paretic	A-F	16.2 (0.8)	22.5 (2.1)	18.5 (1.6)	20.4 (1.6)	21.3 (1.7)	23.7 (0.6)	27.3 (3.5)	22.0 (0.7)	28.7 (3.2)	25.4 (4.0)
		K-F	51.8 (1.6)	76.2 (2.6)	34.7 (1.5)	52.5 (0.9)	51.9(0.6)	62.8 (1.6)	60.0 (1.5)	55.6 (1.9)	68.9 (1.5)	59.6 (0.8)
		H-F	40.2 (1.5)	47.2 (1.8)	37.9 (3.2)	42.7 (0.6)	39.6 (1.2)	48.9 (2.4)	51.4 (5.0)	58.1 (2.1)	48.3 (3.9)	52.4 (0.7)
		H-A	8.1 (1.0)	15.0 (1.3)	12.1 (1.1)	7.9 (0.3)	9.2 (3.1)	7.2 (1.0)	10.2 (1.4)	5.8 (0.5)	18.6 (1.3)	19.3 (1.6)
Variation	Paretic	A-F	−37.2	13.8	−5.4	5.9	−26.2	69.6	79.6	7.0	97.6	−53.1
		K-F	−19.1	18.4	7.2	−8.2	−11.3	−17.4	10.5	3.1	−5.8	−1.6
		H-F	−2.8	−4.4	0.5	−2.8	1.0	−1.1	96.6	−10.0	−18.8	−11.6
		H-A	2.2	−7.9	−2.9	−26.2	75.1	−35.9	−11.5	−14.3	13.5	−21.3
	Non-paretic	A-F	0.4	−35.8	−13.1	−0.1	−8.7	31.0	38.9	29.7	41.1	−34.2
		K-F	−13.8	13.6	2.9	−26.5	−16.3	0.4	15.6	−14.4	−3.4	−12.5
		H-F	2.3	−2.0	−2.2	−7.3	−2.8	0.4	30.4	5.7	−2.5	0.6
		H-A	−18.7	−1.7	−4.3	−34.5	−7.9	−30.2	107.3	−29.7	−4.7	29.8

**Table 3:** Gait Deviation Index for each subject in the baseline and T-FLEX scenarios. The first part is the index for the Paretic (P) and Non-Paretic (N-P) limbs. The lower part indicates the percentage of variation when the participant used the T-FLEX’s actuation system. The highlighted values denote deviation above 10% for both increases (green) and decreases (red).

Subjects		1	2	3	4	5	6	7	8	9	10
Baseline	Paretic	72.0	75.9	69.1	80.2	78.5	80.5	73.3	76.4	67.7	53.8
	Non-paretic	73.0	74.0	57.4	85.3	84.0	101.4	80.8	91.0	64.7	73.2
T-FLEX	Paretic	64.6	61.9	67.2	84.5	67.2	83.0	68.6	78.4	62.6	68.1
	Non-paretic	65.6	60.7	64.7	83.2	85.7	74.3	68.3	60.7	57.9	71.5
Variation	Paretic	−7.3	−14.0	−1.8	2.2	−11.3	2.5	−4.7	2.0	−5.2	14.3
	Non−paretic	−7.4	−13.3	7.3	−2.1	1.7	−27.1	−12.5	−30.3	−6.7	−1.7

**Table 4:** Spatial-temporal parameters and Gait Deviation Index of baseline and assisted gait with T-FLEX actuation system. The highlighted values are parameters with significant changes.

	Baseline			T-FLEX		
	Paretic	Mean	Non-paretic	Paretic	Mean	Non-Paretic
GDI (%)	72.9	-	78.5	70.6	-	69.3
Step length (m)	-	0.9	-	-	0.9	-
Cadence (step/min)	-	99.0	-	-	85.1	-
Walking speed (m/s)	-	0.8	-	-	0.7	-
Stance phase duration (%)	62.9	-	70.0	63.6	-	69.5
Stride length (m)	0.5	-	0.5	0.5	-	0.4
Step width (m)	0.2	-	0.2	0.2	-	0.2

**Table 5:** Percentage of the variation of the spatial-temporal parameters. The highlighted values indicate a change above 10% for both increases (green) and decreases (red).

Subject		1	2	3	4	5	6	7	8	9	10
Paretic	Stance phase duration	7.8	−1.9	1.4	−1.1	3.5	1.1	2.3	−0.8	4.1	−5.9
	Step width	0.0	0.0	0.0	0.1	0.0	0.0	0.0	0.0	0.0	0.0
	Step length	0.1	−0.1	0.0	−0.1	−0.1	−0.1	0.1	−0.1	0.1	0.0
Non-paretic	Stance phase duration	1.6	−5.3	0.0	2.6	−2.1	5.3	−4.1	0.6	−1.4	−1.7
	Step width	0.0	0.0	0.0	0.0	0.0	0.0	0.0	0.0	0.0	0.0
	Step length	0.0	−0.1	0.0	0.0	0.0	−0.1	0.2	−0.1	0.0	−0.1
Walking speed		0.0	−0.1	0.0	−0.3	−0.2	0.0	0.2	−0.3	−0.1	−0.1
Stride length		0.0	−0.1	0.0	−0.2	0.0	−0.1	0.4	−0.2	0.0	0.0
Cadence		−11.0	2.0	−6.1	−15.0	−21.1	−24.0	−11.6	20.0	−17.8	−14.7

**Table 6:** The probability value (*p*-value) of each subject for the stance and swing phases. The highlighted cells indicate a statistical difference (*p* < 0.05) calculated through Student’s *t*-tests.

Subjects	Stance Phase	Swing Phase
1	$1.41\times 10^{-4}$	$4.38\times 10^{-11}$
2	$3.10\times 10^{-3}$	$8.54\times 10^{-1}$
3	$1.40\times 10^{-3}$	$2.85\times 10^{-1}$
4	$1.03\times 10^{-9}$	$1.18\times 10^{-10}$
5	$5.27\times 10^{-2}$	$1.67\times 10^{-23}$
6	$8.12\times 10^{-3}$	$3.21\times 10^{-2}$
7	$8.89\times 10^{-1}$	$8.26\times 10^{-2}$
8	$7.47\times 10^{-1}$	$1.65\times 10^{-8}$
9	$3.90\times 10^{-15}$	$1.08\times 10^{-33}$
10	$8.10\times 10^{-2}$	$1.14\times 10^{-12}$

## Data Availability

This study was registered as Preliminary Biomechanical and Usability Study of an Active Ankle-Foot Orthesis for Stroke Survivors on 30 January 2020 in Clinical Trials with the identifier No NCT04249349 (available at https://clinicaltrials.gov/ct2/show/NCT04249349) (accessed on 30 January 2020).

## Sample Availability

The data that support the findings of this study are openly available in Figshare at https://doi.org/10.6084/m9.figshare.12576965.v1 (accessed on 27 June 2020).

## Abbreviations

The following abbreviations are used in this manuscript:

PAFO	Powered Ankle-Foot Orthosis
ADLs	Activities of Daily Living
AFO	Ankle-Foot Orthosis
A-F	Dorsi-plantarflexion
FSR	Force Sensitive Resistor
F-R	Foot Rotation
GDI	Gait Deviation Index
GPS	Gait Profile Score
H-A	Hip Adduction-Abduction
H-F	Hip Flexo-Extension
K-F	Knee Flexion-Extension
MAP	Movement Analysis Profile
P-A	Pelvis Anterior-posterior
P-R	Pelvic Rotation
P-U	Pelvic Up-down
ROM	Range of Motion
TO	Toe-Off
